# Old Is (Not) Gold: Midazolam Monotherapy versus Midazolam Plus Fentanyl for Sedation during Cardiac Catheterization

**DOI:** 10.1155/2021/9932171

**Published:** 2021-08-02

**Authors:** William Black, Raj Baljepally, Laylan Shali, Omar Alsharif, Scott Warden, Eric Heidel, Xiaopeng Zhao

**Affiliations:** ^1^University of Tennessee Medical Center, Heart Lung Vascular Institute, Department of Cardiology, Knoxville, TN, USA; ^2^Department of Mechanical, Aerospace, and Biomedical Engineering, University of Tennessee, Knoxville, TN, USA

## Abstract

**Objective:**

We aimed to study the differences in perception of pain during cardiac catheterization with midazolam monotherapy compared to the current standard of midazolam plus fentanyl.

**Background:**

Procedural sedation is important to ensure comfort and safety in patients undergoing left heart catheterization. Despite the widespread use of midazolam and fentanyl for procedural sedation, the effectiveness of this dual agent approach to sedation has never been studied in comparison to midazolam monotherapy.

**Methods:**

A total of 129 patients undergoing sedation for outpatient elective cardiac catheterization were randomly assigned to either midazolam monotherapy (*n* = 69) or combination of midazolam and fentanyl (*n* = 60). The primary outcome was assessment of pain perception prior to discharge by patient completion of a pain questionnaire. Participants were asked if they experienced any pain during their procedure (yes/no) and, if yes, asked to rate their overall pain level using a 10-point Likert scale that ranged from 1 (minimal pain) to 10 (worst pain imaginable).

**Results:**

Most patients (*n* = 94, 73%) reported no pain during their procedure. Patients sedated with midazolam monotherapy reported similar average pain scores compared to patients sedated with the combination of midazolam and fentanyl (1.1 vs. 1.1, *p*=0.95).

**Conclusions:**

Among patients undergoing elective cardiac catheterization, no significant differences in pain scores were noted between sedation with midazolam alone compared to midazolam and fentanyl. Due to fentanyl's unfavorable interaction with P2Y12 agents, increased costs, and addiction potential, it is imperative that cardiologists revisit the role of effective procedural sedation with a single agent and avoid the use of fentanyl.

## 1. Introduction

The administration of procedural sedation is a common practice to promote safety and ensure patient comfort during cardiac catheterization procedures [1, 2]. The Society for Cardiovascular Angiography and Interventions expert consensus statement on best practices in the cardiac catheterization laboratory encourages the use of moderate sedation for patients undergoing coronary angiography [1]. Intravenous midazolam and fentanyl have been used historically to achieve adequate sedation for cardiac catheterization procedures, and this dual therapy has remained the mainstay of procedural sedation in the cardiac cath lab as well as the electrophysiology lab [3–6]. Despite the widespread use of this practice, there is little clinical evidence evaluating the effectiveness of these medications for patients undergoing catheterization procedures. The rate of sedation usage for cardiac procedures varies widely and may be influenced by culture, patient expectations, training, and geography [7]. An international survey found that 92% of cardiologists in North America reported using sedation during cardiac catheterization compared to just 38% of cardiologists in other countries [8]. The same study found that a majority of US cardiologists (60%) perceived that 75–100% of their patients would want sedation, while a majority of European cardiologists (60%) perceived that only 0–25% of their patients would want sedation.

Opioids have been the cornerstone of analgesia for both chronic and acute pain since the late 1980s as part of a push by the Joint Commission to label pain as “the fifth vital sign” [9]. The rise in opioid addiction is an unintended consequence of this campaign and is now a major public health crisis facing the United States [10, 11]. In 2016 alone, more than 42,0000 Americans died from opioid overdose, and the annual number of opioid deaths is projected to reach nearly 82,000 by 2025 [12, 13]. There is mounting evidence that the risk of opioid dependence increases with even a single opioid exposure, and many heroin addicts were first exposed to opioids by a legitimate medical prescription [14, 15]. A recent retrospective cohort study found that opioid-naive patients who were prescribed opioids in the emergency department for acute pain are at an increased risk for additional opioid use at one year compared to patients who were not prescribed opioids [16, 17]. Despite the potential for postprocedural opioid use, preprocedural screening for a prior history of substance abuse is not routinely performed by cardiologists prior to catheterization [18].

In addition to the potential for opioid abuse, the use of opioid medications may interfere with the absorption of P2Y12 inhibitors due to delayed gastric emptying. Rapid absorption of P2Y12 inhibitors is critical for patients with acute coronary syndrome and patients undergoing percutaneous coronary intervention to prevent acute stent thrombosis [19, 20]. The PACIFY trial demonstrated that fentanyl delayed the absorption of ticagrelor by up to four hours and resulted in attenuated platelet inhibition at two hours [21]. Interestingly, there was no significant difference in average patient comfort between the fentanyl and no-fentanyl arms in that study (all patients received local anesthetic and midazolam).

Several small studies have demonstrated adequate sedation for catheter-based procedures with either minimal sedation or benzodiazepine monotherapy [22–24]. The combination of benzodiazepines and opioids has been shown to reduce the risk of radial artery spasm and improve patient tolerability [25]. Intraarterial vasodilators such as nitroglycerin and verapamil are routinely administered to reduce radial artery spasm, so it is unclear whether the addition of fentanyl is necessary to prevent radial artery spasm or if sedation with benzodiazepines alone would be adequate. We sought to compare the differences in pain perception with midazolam alone versus the current standard of care of midazolam plus fentanyl during cardiac catheterization.

## 2. Methods

### 2.1. Study Design

This is a single-center, single-blinded randomized prospective study performed at the University of Tennessee Medical Center, Knoxville, TN, USA. The study was approved by the institutional review board (IRB).

### 2.2. Study Population

Patients aged 18 years and older who were referred for outpatient elective cardiac catheterization (CC) including left heart catheterization (LHC), right heart catheterization (RHC), and LHC with coronary angiography from July 2019 to September 2020 were eligible for inclusion in the study. Patients were excluded if they had a known allergy to one or both of the study medications.

### 2.3. Procedural Details

Informed consent was obtained, and patients were randomly assigned to two groups: midazolam monotherapy (*n* = 69) and midazolam plus fentanyl (*n* = 60). The randomization sequence was computer generated and concealed in sealed opaque envelopes. Patients, but not CC operators, were blinded to the study medications. Patients in the midazolam group were given intravenous midazolam with dosages based on age, gender, and body mass (0.5–4 mg) in accordance with normal pharmacological practice. Patients in the midazolam and fentanyl groups were given intravenous midazolam (0.5–4 mg) plus intravenous fentanyl (25–125 mcg) with fentanyl similarly dosed based on age, gender, and body mass. Patients from the midazolam group that required additional sedation were given a second dose of midazolam. If adequate sedation could not be achieved with midazolam alone, they were allowed to crossover and receive fentanyl. In all patients, local anesthetic (1% xylocaine) was administered at the access site after administration of sedation. Oral diazepam (5–10 mg) and diphenhydramine (25 mg) were given 30 minutes before commencement of the procedure unless patients were allergic to these medications. After the procedure, patients who underwent femoral arterial access were positioned supine and flat for at least 2 hours (longer if no closure device was deployed). Patients who underwent catheterization via the right radial artery had a radial compression bracelet placed and were allowed to sit up after returning to the recovery room. Patients were administered a pain survey by a research assistant to complete at the time of discharge (3 hours after the completion of the procedure for radial access and 6 hours after the procedure for femoral access).

Participants were asked if they experienced any pain during their procedure (yes/no) and, if yes, asked to rate their overall pain level for the periprocedural period using a 10-point Likert scale that ranged from 1 (minimal pain) to 10 (worst pain imaginable). Patients that had undergone prior LHC were asked to compare their level of pain to prior LHC (less pain, the same, or more pain).

### 2.4. Statistical Analysis

We hypothesized a medium effect size, *d* = 0.5, associated with the difference in treatment modalities. Using the aforementioned effect size, a two-sided hypothesis, an alpha value of 0.05, a beta value of 0.20, and an equal allocation ratio to treatment arms (1 : 1), it was found that *n* = 128 participants would be needed in the study to achieve adequate statistical power to detect a difference if it actually existed in the population. The sample size calculation was performed using G∗Power Version 3.1.

Statistical analysis was performed using chi-square analyses to compare the midazolam and midazolam plus fentanyl groups on categorical variables. Frequency and percentage statistics were used to describe the statistical findings of the chi-square statistics. Independent-samples *t*-tests were used to compare the groups on continuous variables. Means and standard deviations were reported and interpreted for the *t*-test findings. Statistical significance was assumed at an alpha value of 0.05, and all analyses were performed using SPSS Version 26 (Armonk, NY: IBM Corp.).

## 3. Results

**Figure 1:**
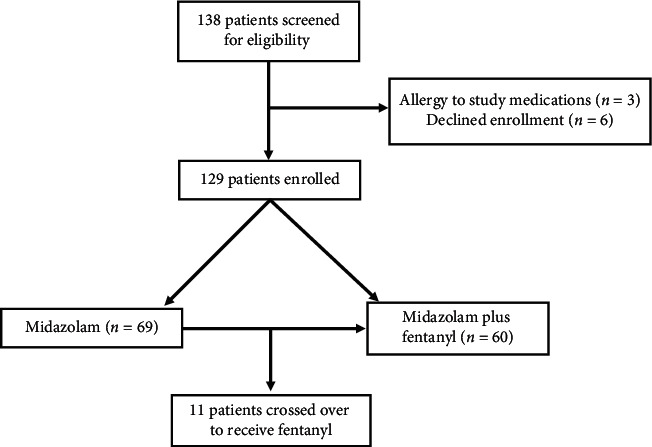
Flowchart of patient enrollment.

A total of 129 patients were enrolled and completed the patient pain questionnaire (Figure 1). Participants aged from 32 to 85 years (mean 65.6, standard deviation 10.8). The majority (83%, *n* = 107) were classified as overweight or obese according to their body mass index (≥25 kg/m^2^). The baseline demographics of the study population are listed in Table 1. The majority of patients in each group underwent radial access and had LHC performed as indicated.

In general, patients reported a low level of discomfort during the procedure. The percentage of patients who experienced any pain was similar between the midazolam and midazolam plus fentanyl groups (26.1% vs. 28.3%, *p*=0.78). The average pain scores were identical between the two treatment groups as indicated in Table 2 (1.1 vs. 1.1, *p*=0.95). Nearly half of the patients had undergone prior LHC (*n* = 63, 49%). Twenty-seven patients reported similar pain compared to their last LHC, 30 reported less pain, and 6 patients reported more pain, which did not statistically differ between the two groups. Of the 69 patients in the midazolam monotherapy group, only 16% (*n* = 11) crossed over and received fentanyl.

There were 6 patients in each treatment group who reported a history of chronic pain. Patients with a history of chronic pain who received only midazolam reported higher average pain scores than patients with chronic pain who received both midazolam and fentanyl (2.83 vs. 0.5, *p*=0.037). Patients who reported pain during their procedure had longer average procedure times compared to patients who did not report any pain (52.51 minutes vs. 35.84 minutes, *p*=0.006). The rates of pain did not statistically differ based on artery accessed (31.6% for femoral access, 25.3% for radial access, *p*=0.46%). Of the patients who did report pain, pain scores did not differ based on arterial access site (1.21 for femoral access, 1.01 for radial access, *p*=0.63).

## 4. Discussion

Sedation is desirable during catheter-based cardiac procedures to produce analgesia and to improve overall patient comfort [26]. Despite the prevalence of coronary angiography, there is a paucity of data regarding optimal sedation techniques for this procedure. The purpose of this study was to examine the effectiveness of sedation with midazolam alone versus the combination of midazolam and fentanyl in patients undergoing elective CC. Patient comfort was evaluated after the procedures by completion of a pain questionnaire prior to discharge. Most patients reported no pain at all during their procedure. Of the 35 patients who did experience pain during their procedure, 28 reported their pain to be less than 5 on a scale from 1 to 10. For the primary endpoint of patient reported periprocedural pain, there was no difference in midazolam alone compared to midazolam and fentanyl combination therapy. Our study demonstrates that midazolam monotherapy is an acceptable choice for procedural sedation for CC.

There are clinical scenarios where an initial sedation strategy with midazolam and fentanyl may still be utilized. Although we enrolled few patients with a history of chronic pain (*n* = 12), patients with chronic pain reported lower average pain scores when they received both fentanyl and midazolam for sedation (0.5 vs. 2.83, *p*=0.037). Longer cases may benefit from sedation with midazolam and fentanyl as well. Patients who reported pain had an average procedure length of 52.51 minutes (SD = 22.87 minutes) compared to 35.84 minutes (SD 43.91) in patients who did not report pain (*p*=0.006). Musculoskeletal pain, particularly lumbar pain, is the most common site of pain reported during left heart catheterization [27]. Although we did not ask patients to localize their pain, we suspect that patients with longer procedural times experienced more musculoskeletal pain due to discomfort from the hard cath table. There was no significant difference in average reported pain scores in patients who underwent femoral arterial access compared to radial arterial access (1.21, SD = 2.01 vs. 1.01, SD = 2.15, *p*=0.63).

There are several potential benefits to using midazolam monotherapy for procedural sedation. First, it has been well-demonstrated that fentanyl administration lowers plasma concentrations of ticagrelor and delays its antiplatelet effects as demonstrated in the PACIFY trial [21]. Morphine reduces absorption and impairs P2Y12 inhibition for all P2Y12 agents [21, 28, 29]. Second, the potential cost savings of avoiding unnecessary fentanyl use is certainly important in this era of cost containment. Finally, and most importantly, the ever-increasing opioid epidemic is a major US health crisis with more than 42,000 Americans dying of opioid overdose each year [11, 12]. As previously mentioned, the risk for dependence occurs not only with increasing the frequency of exposure but even with a single exposure to opioid medications [14, 15]. For opioid-naïve patients, clinicians should strive to keep them opioid-naïve [30]. We believe that these facts present a compelling argument for limiting the use narcotics for sedation during routine elective CC.

### 4.1. Limitations

The primary limitations of our study include smaller sample size, single center, and single-blinded randomization. Other limitations involving the study design include patient reporting on a questionnaire, crossover allowance from midazolam monotherapy to midazolam and fentanyl, and more females in the midazolam plus fentanyl group. These limitations are minimized by the outcomes observed. Assessment of pain relied upon patient reporting postprocedure, which is subjective, and each patient may have a different pain tolerance. Eventhough our study design allowed for crossover, patients in the midazolam group rarely crossed over to receive fentanyl for sedation (17%, *n* = 12).

## 5. Conclusion

Midazolam monotherapy produces similar pain perception compared to a combination of midazolam and fentanyl during sedation during cardiac catheterization. The combination of midazolam and fentanyl has historically been the standard regimen for sedation in the catheterization lab. However, it may be important to consider midazolam monotherapy as an alternative. We believe a larger, randomized multicenter study is needed to further validate our findings. This is an important issue for multiple reasons including fentanyl's documented inhibition of absorption and efficacy of P2Y12 agents, potential cost savings of reducing fentanyl use, and reducing potential for opioid addiction. This study suggests that midazolam monotherapy is a reasonable option for sedation for cardiac catheterization.

## Figures and Tables

**Table 1:** Baseline characteristics by group.

Variable	M *N* = 69	M + F *N* = 60	*P* value
Age^*∗*^	66.17 (10.18)	63.87 (12.70)	0.50
BMI^*∗*^	30.03 (6.86)	32.03 (6.42)	0.09
Gender (female), *n* (%)^*∗∗*^	15 (21.74%)	26 (43.33%)	0.008^*∗∗∗*^
CAD^*∗∗*^	38 (55.07%)	27 (45%)	0.44
CHF^*∗∗*^	10 (14.49%)	6 (10%)	0.44
HLD^*∗∗*^	51 (73.91%)	40 (66.67%)	0.37
DM^*∗∗*^	17 (24.64%)	19 (31.67%)	0.38
PVD^*∗∗*^	5 (7.25%)	3 (5%)	0.60
HTN^*∗∗*^	55 (79.71%)	49 (81.67%)	0.78
Tobacco use^*∗∗*^	23 (33.33%)	13 (21.67%)	0.14
CKD^*∗∗*^	8 (11.59%)	4 (6.67%)	0.34
UA^*∗∗*^	2 (2.90%)	1 (1.67%)	0.65
Chronic pain^*∗∗*^	6 (8.70%)	6 (10%)	0.80

^*∗*^Values are mean (standard deviation). ^*∗∗*^Values are frequency (percentage). ^*∗∗∗*^, *P* < 0.05. M, midazolam group; M + F, midazolam plus the fentanyl group.

**Table 2:** Comparison of independent groups on primary and secondary endpoints.

Outcome	M *N* = 69	M + F *N* = 60	Mean difference (95% CI)	*P* value
Pain during procedure, *n* (%)^*∗∗*^	18 (26.09%)	17 (28.33%)	—	0.78
Pain severity (1–10)^*∗*^	1.1 (2.0)	1.1 (2.3)	−0.25 (−0.77–0.72)	0.95
Premidazolam (mg)^*∗*^	1.73 (1.02)	1.59 (0.96)	0.14 (−0.21–0.49)	0.43
Prefentanyl (mg)^*∗*^	0 (0)	50 (25)	—	0^†^
Total midazolam (mg)^*∗*^	3.71 (2.02)	3.43 (1.93)	0.28 (−0.42–0.97)	0.43
Total fentanyl (mg)^*∗*^	0 (0)	75 (50)	—	0^†^
Total procedure time (mins)^*∗*^	41.88 (34.40)	38.62 (26.19)	3.27 (−7.51–14.05)	0.55

^*∗*^Values are mean (standard deviation). ^*∗∗*^Values are frequency (percentage). ^†^*P* < 0.05. M, midazolam group; M + F, midazolam plus fentanyl group. ^†^*P* value could not be calculated as fentanyl was not provided in the midazolam only group.

## Data Availability

The data used to support the findings of this study are available from the corresponding author upon request.
